# Interdisciplinary Management of a Skeletal Class III Malocclusion With Impacted Central Incisor and Lateral Incisor Revascularization: A Case Report

**DOI:** 10.7759/cureus.91768

**Published:** 2025-09-07

**Authors:** Yosra Tabchi, Abdoul Hafizou Rabé Amani, Fatima Zaoui

**Affiliations:** 1 Dentistry, Sidi Mohamed Ben Abdellah University of Fez, Fez, MAR; 2 Odontology, Niamey Military Hospital, Niamey, NER; 3 Orthodontics and Dentofacial Orthopedics, Mohammed V University in Rabat, Faculty of Dental Medicine, Rabat, MAR

**Keywords:** biomechanics in orthodontics, case report, class iii malocclusion, impacted central incisor, multidisciplinary approach, orthodontics treatment

## Abstract

Impacted maxillary incisors are rare and pose significant esthetic and functional challenges, particularly when associated with other dental anomalies and skeletal discrepancies. This case highlights the interdisciplinary management of such a complex case.

An 18-year-old male presented with a missing maxillary left central incisor, a fractured maxillary right lateral incisor of uncertain prognosis, and a skeletal Class III malocclusion with anterior and lateral crossbite. Clinical examination, panoramic radiography, and cone beam computed tomography (CBCT) confirmed impaction of the maxillary left central incisor, presence of periapical pathology related to the lateral incisor, and a skeletal Class III pattern.

Treatment included regenerative endodontic therapy for the lateral incisor, surgical exposure of the impacted central incisor using the closed-eruption technique, and biomechanically controlled orthodontic traction. Anchorage was reinforced with a transpalatal bar, and gradual third-order bends were applied for root torque control.

The impacted central incisor was successfully aligned into the arch, the lateral incisor was preserved, and functional occlusion with corrected overjet and overbite was achieved. The treatment duration was 30 months, and bonded retainers were placed. The patient reported high satisfaction with both functional and esthetic outcomes.

This case demonstrates how a patient-centered, multidisciplinary approach can yield predictable, stable, and esthetically pleasing results in the management of impacted maxillary incisors with concomitant anomalies.

## Introduction

Skeletal Class III malocclusion has long been considered the most challenging malocclusion to treat. This discrepancy arises from maxillary retrognathism, mandibular prognathism, or a combination of both. Depending on the severity, treatment options range from orthodontic camouflage to combined orthodontic and orthognathic surgery approaches. Furthermore, management becomes even more complex in cases associated with additional dental anomalies, such as impacted or missing anterior teeth, which can compromise alveolar growth and further complicate treatment planning [1,2].

According to the French Orthodontic Society, a tooth is considered impacted when it remains retained within its follicular sac, without communication with the oral cavity, for at least two years beyond its normal eruption date. The impaction of maxillary incisors, although rare, with a reported incidence ranging from 0.13% to 2.6%, requires a multidisciplinary treatment plan. Eruption failure may occur due to pathological obstructions along the eruptive path, tooth malformation, or dilacerations. Treatment options range from conservative to radical approaches. Studies report that the prognosis of orthodontic-surgical treatment of impacted incisors is generally good, with failures associated with severe dilacerations [3-5].

This case report illustrates the interdisciplinary treatment of a combination of skeletal Class III malocclusion, an impacted maxillary central incisor, and a traumatized lateral incisor. The case shows the importance of coordinated orthodontic, surgical, and endodontic clinical management to achieve predictable functional and esthetic objectives.

## Case presentation

This case report has been prepared in accordance with the CARE (Case Report) guidelines.

Patient information

An 18-year-old male patient presented with the chief complaint of a missing maxillary left central incisor. He had previously visited a dental clinic to treat a fractured maxillary right lateral incisor resulting from a previous traumatic injury; however, during the examination, the dentist incidentally noted the presence of an impacted maxillary left central incisor. Without initiating any treatment, the dentist referred the patient to our department for multidisciplinary care. The patient had no significant medical history. Family history revealed no similar maxillofacial deformities.

Clinical findings

**Figure 1:**
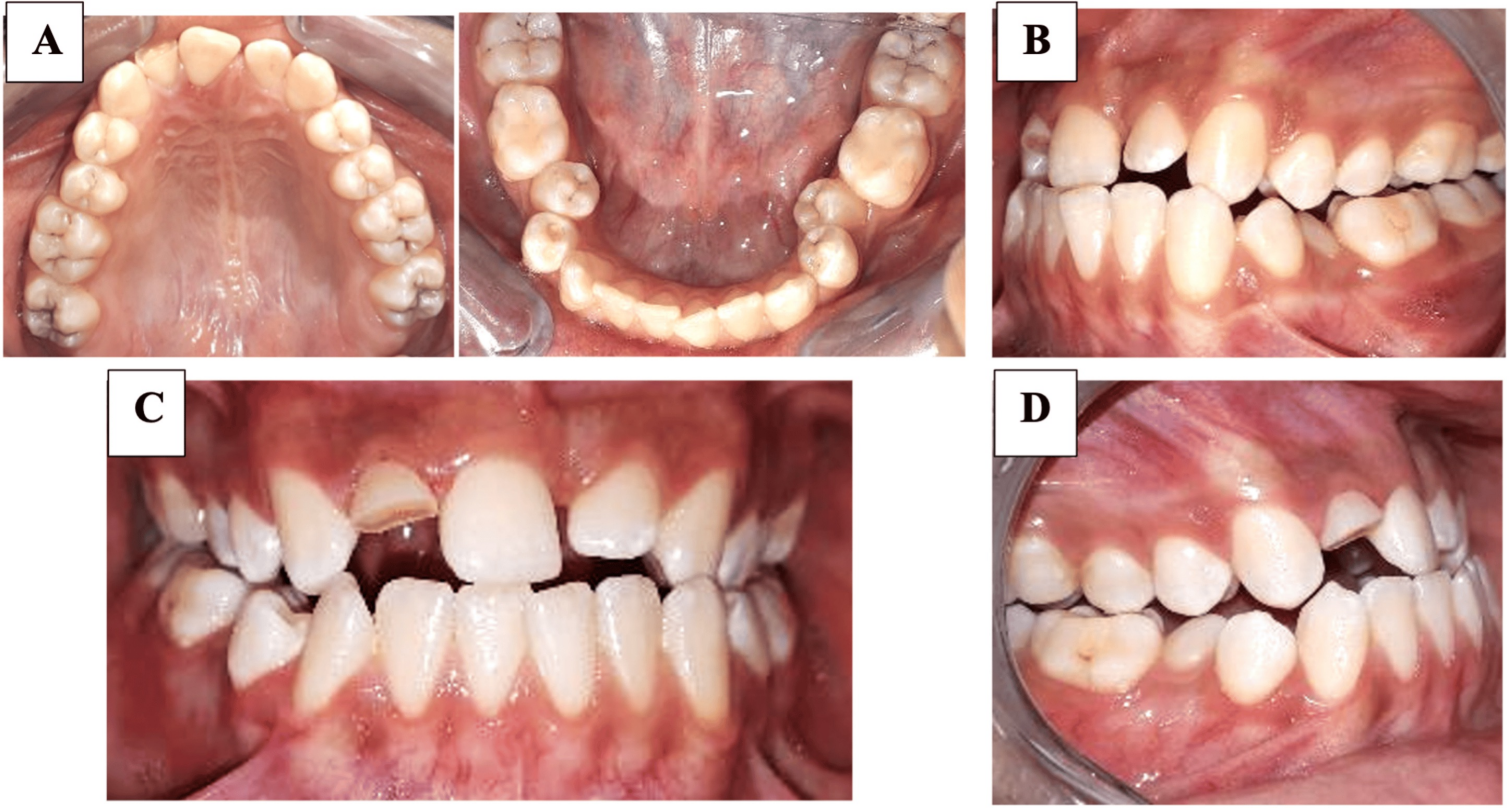
Pre-treatment intraoral photographs (A) Occlusal view of the maxillary and mandibular arch, (B): Left occlusal view, (C): Front occlusal view, (D): Right occlusal view

The intraoral examination showed fair oral health, with signs of gingivitis. The maxillary dental arch showed the absence of the upper left central incisor, a fractured upper right lateral incisor, and a slight rotation of the upper first molar. The mandibular arch displayed 8 mm of crowding in the lateral segments (canine-premolar region). The inter-arch relationship showed an anterior and lateral crossbite, with a full Class III molar on both the right and left sides. The maxillary midline was shifted to the left by 3 mm. Figure 1 shows intraoral photographs.

Diagnostic assessment

The panoramic radiograph confirmed the impaction of the upper left central incisor, associated with pathological obstructions blocking the eruptive path of the permanent tooth. The CBCT scan revealed the vestibular position of the tooth and its proximity to the apex of the upper right central incisor. The panoramic radiograph also showed the presence of all third molars, and a periapical bone resorption related to the upper right lateral incisor.

Cephalometric examination revealed a skeletal class III malocclusion with a hypodivergent growth pattern and proclined maxillary incisors. 

**Figure 2:**
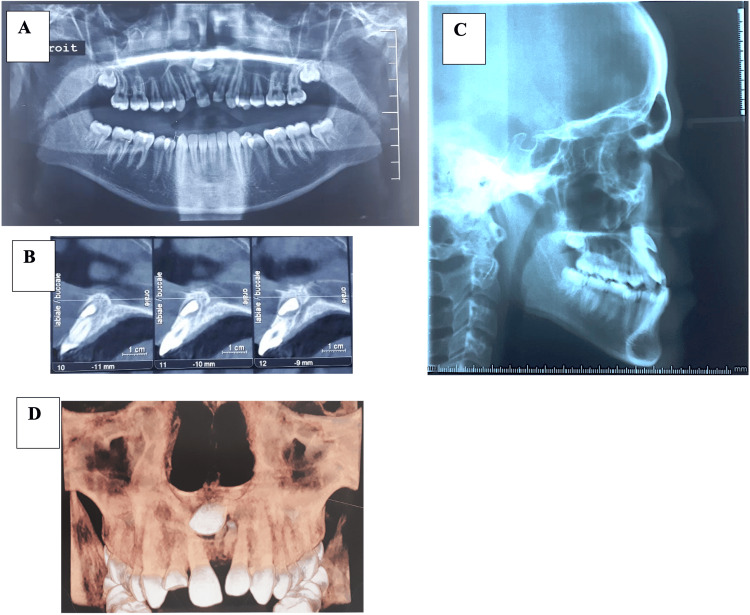
Pre-treatment radiographs (A): Panoramic radiograph, (B): CBCT scans: Cross-sectional slices of the impacted tooth, (C): Lateral cephalometric radiograph, (D): CBCT scan: 3D reconstruction of the maxillary region CBCT: cone beam computed tomography

Table 1 summarizes the pretreatment cephalometric examination of our patient. Figure 2 shows the pre-treatment radiographs of the patient.

**Table 1:** Pretreatment cephalometric variables of the patient Cephalometric variables were measured in millimeters (mm) and degrees (°) using standard angular and linear analysis protocols.

Cephalometric variables	Pretreatment
Maxilla SNA (°)	75
Mandible SNB (°)	77
Maxilla-mandible: ANB (°)	-2
Maxilla-mandible: AoBo (mm)	-5
Vertical relation: Sn-GoGn (°)	25
Vertical relation: FMA (°)	23
Maxillary dental parameters: I to Na (mm)	6
Maxillary dental parameters: I to Na (°)	32
Mandible dental parameters: i to Nb (mm)	4
Mandible dental parameters: i to Nb (°)	24
Mandible dental parameters: IMPA (°)	88
Maxillary-mandible: Interincisal-angle (°)	127

Treatment plan

Pre-orthodontic Phase

Before initiating orthodontic treatment, initial periodontal therapy was performed to ensure a healthy gingival condition. In addition, the patient received oral hygiene instructions to maintain optimal periodontal health throughout treatment.

Moreover, the fractured upper right lateral incisor was treated by our fellow dentist by performing pulp revascularization treatment to promote apical root development of the immature permanent tooth and restore pulpal vitality. An intracanal medicament was placed and left in the canal for four weeks. Later, the antibiotic was removed, and periapical bleeding was induced to form an apical blood clot, serving as a scaffold for tissue regeneration.

The patient was monitored clinically and radiographically at 3, 6, and 12 months, and orthodontic treatment was initiated only after the absence of symptoms and radiographic evidence of apical closure of the tooth was obtained.

Orthodontic Treatment

Orthodontic treatment was initiated before the surgical procedure, using a 0.022’’ McLaughlin Bennett Trevisi (MBT) preadjusted edgewise appliance. The maxillary and mandibular arches were aligned using 0.016’’ nickel-titanium (NiTi) archwires, followed progressively by heavier archwires, including 0.017x0.025’’ and 0.019x0.025’’ stainless steel.

Before surgical exposure, an open coil spring was used to create space for the impacted maxillary incisor. Anchorage was reinforced with a transpalatal bar, which was also used to derotate the maxillary molars, and an orthodontic traction arm was placed palatally. During surgery, the pathological obstructions were removed, and a vestibular button was bonded to the exposed surface of the central incisor. The direction of traction was planned to move the tooth away from the root of the adjacent central incisor. Traction force was applied using an elastomeric chain, and a 0.019x0.025’’ stainless steel base archwire, with adjacent teeth consolidated using a ligature wire.

Once the central incisor approached the occlusal plane, a bracket was bonded to it, and alignment was continued using 0.016” NiTi, 0.017x0.025” NiTi, and 0.017x0.025” stainless steel. During the finishing phase, a radiculo-palatal torque was applied to the left tractioned incisor on a 0.019x0.025’’ stainless steel archwire to correct its inclination.

In the mandibular arch, the right and left first mandibular premolars were extracted to resolve crowding, achieve a correct overjet, and obtain a proper overbite.

**Figure 3:**
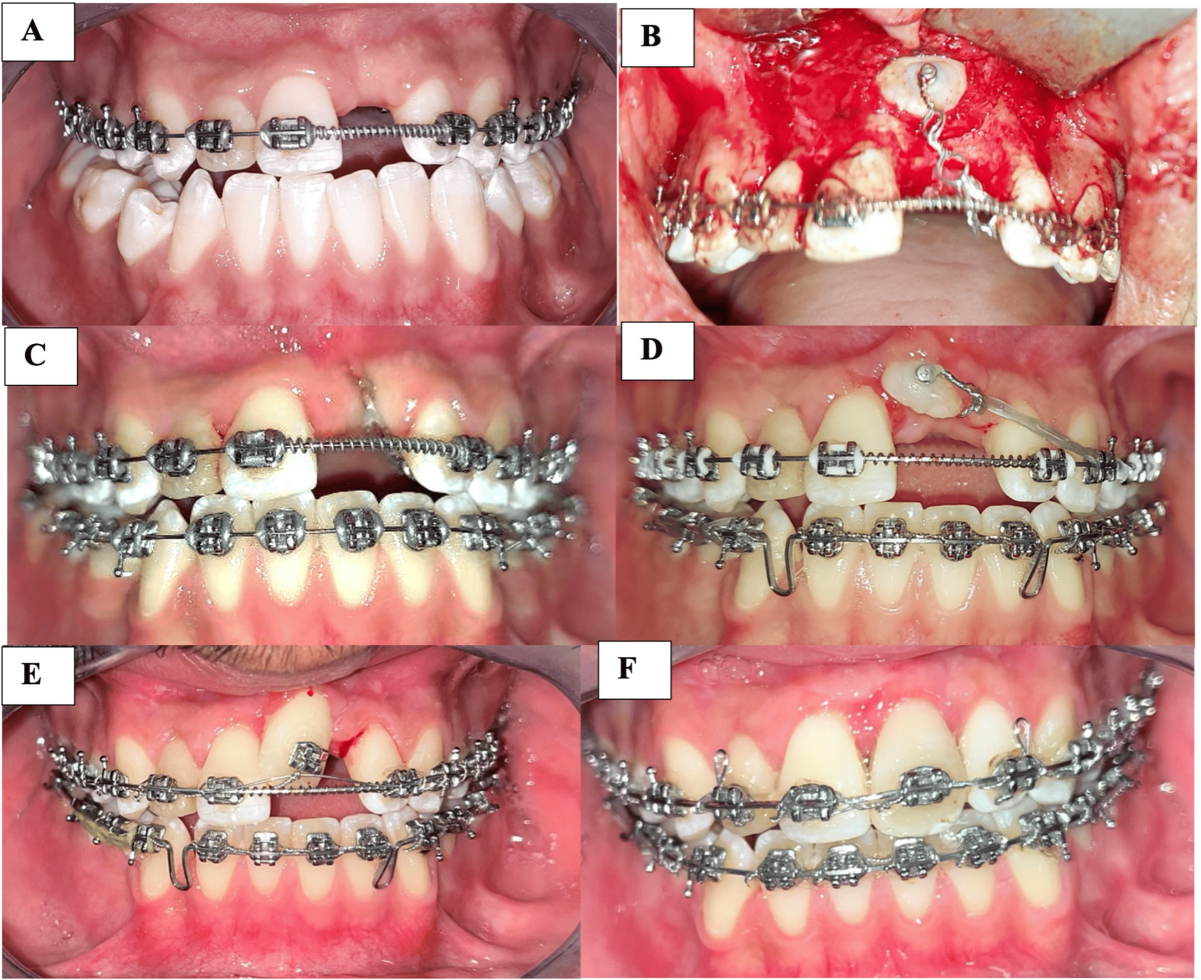
Orthodontic treatment of the impacted maxillary left incisor A: Creating space for the impacted tooth, B: Surgical exposure, C: Traction, D: Redirection of the impacted tooth, E: Aligning the impacted tooth, D: Finishing phase

Figure 3 shows the resumption of the orthodontic process of managing the impacted maxillary incisor.

Follow-up and Outcomes

Our treatment objectives were achieved. The patient was satisfied with the preservation of his natural teeth and the improvement in his smile and bite. The case was finalized with a therapeutic Class III occlusion, with necessary occlusal finishing to stabilize the results. We obtained a perfect coincidence of the dental midlines, along with a correct overjet and overbite; the lateral crossbite was corrected. The maxillary third molars are scheduled for extraction.

Long-term follow-up data are not available; however, the patient remains under observation, and stability will continue to be monitored.

**Figure 4:**
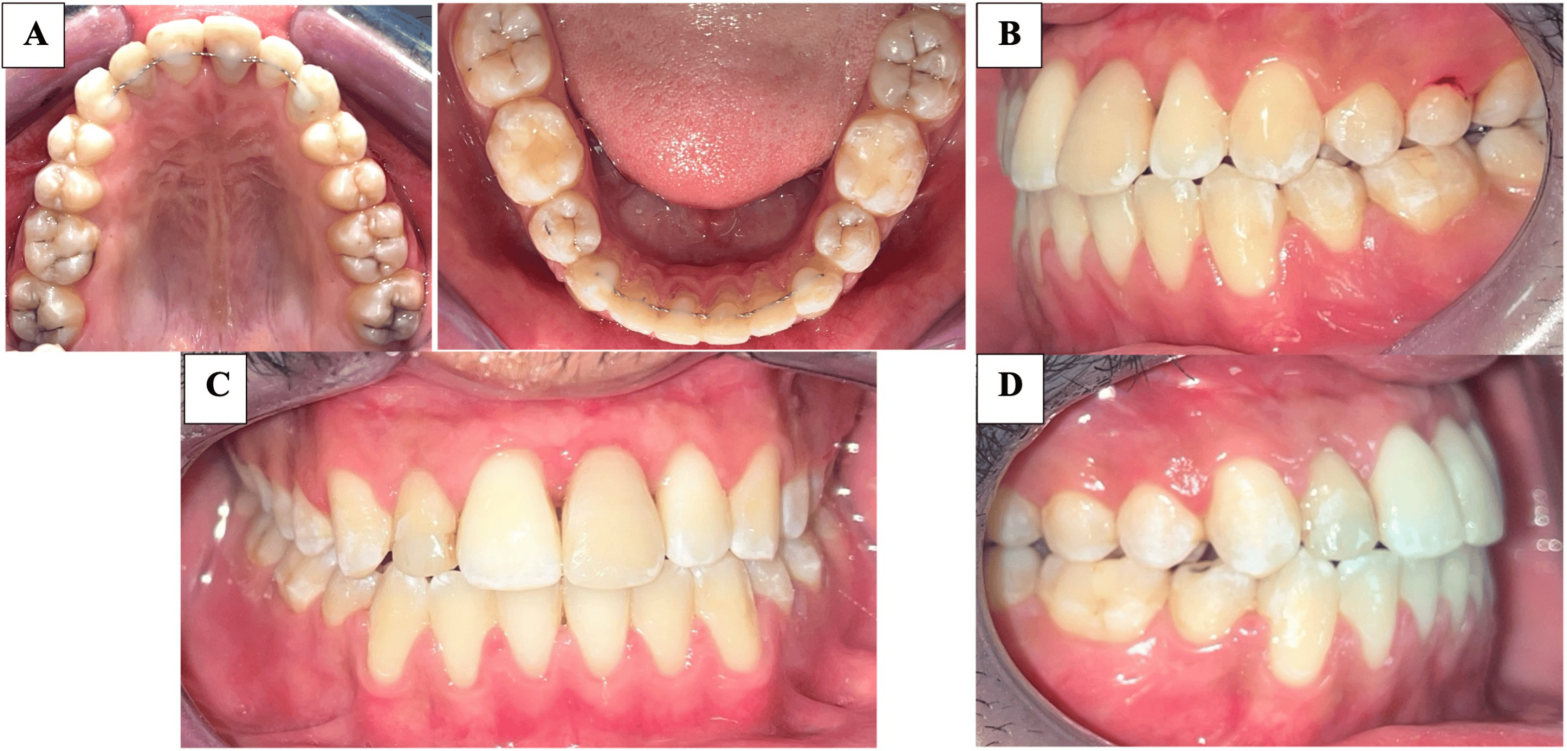
Post-treatment intraoral photographs (A) Occlusal view of the maxillary and mandibular arch, (B): Left occlusal view, (C): Front occlusal view, (D): Right occlusal view

**Figure 5:**
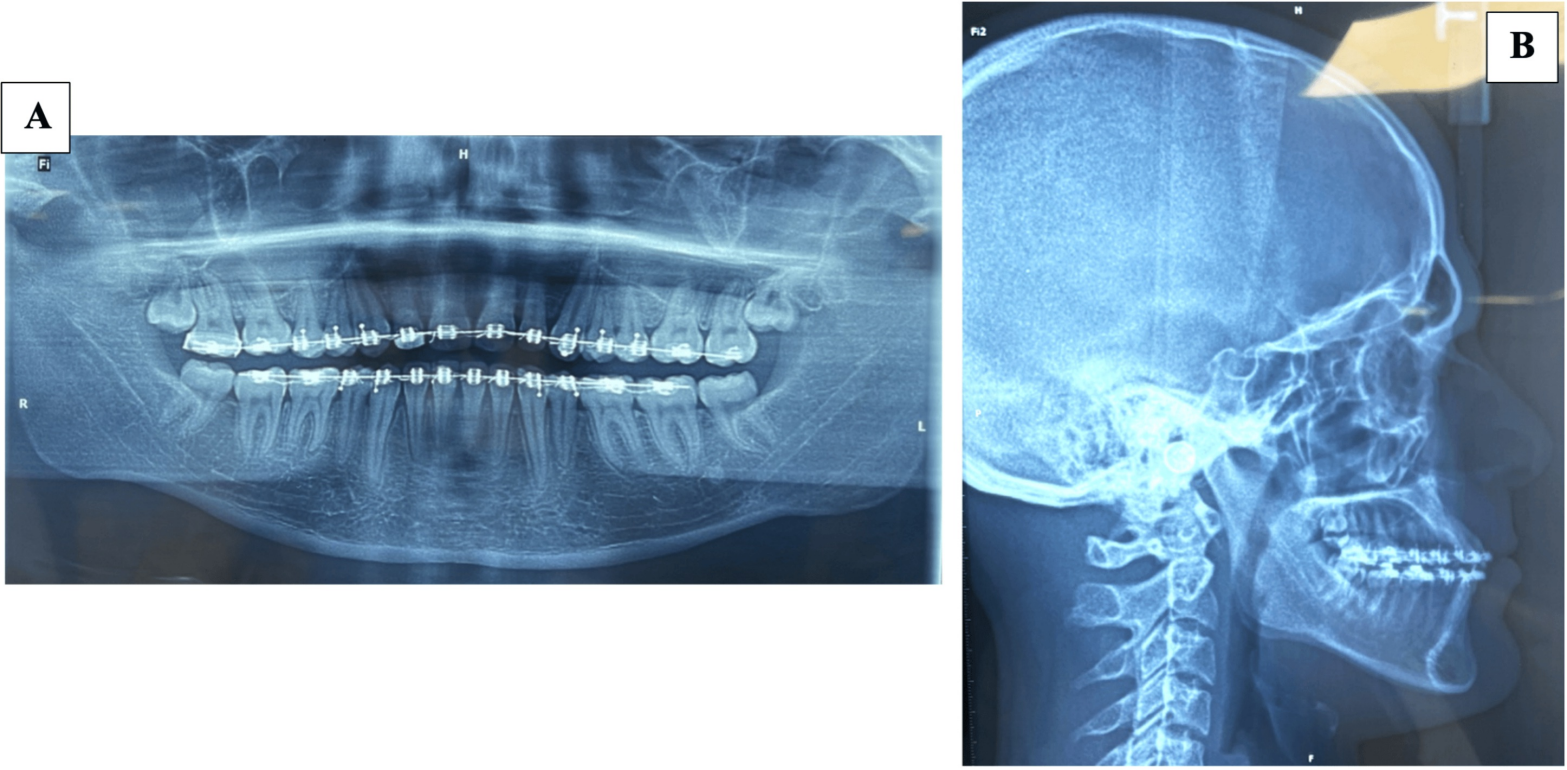
Post-treatment radiographs (A) Panoramic radiograph, (B): Lateral cephalometric radiograph

Figure 4 shows post-treatment intraoral photographs, while Figure 5 shows post-treatment radiographs of the patient.

The cephalometric changes shown in Table 2 were mainly dental, reflecting the correction of the position and inclination of both maxillary and mandibular incisors, with good skeletal vertical control.

**Table 2:** Cephalometric summary Cephalometric variables were measured in millimeters (mm) and degrees (°) using standard angular and linear analysis protocols.

Cephalometric variables	Pretreatment	Post-treatment
Maxilla SNA (°)	75	75
Mandible SNB (°)	77	77
Maxilla-mandible: ANB (°)	-2	-2
Maxilla-mandible: AoBo (mm)	-5	-5
Vertical relation: Sn-GoGn (°)	25	27
Vertical relation: FMA (°)	23	24
Maxillary dental parameters: I to Na (mm)	6	5
Maxillary dental parameters: I to Na (°)	32	30
Mandible dental parameters: i to Nb (mm)	4	5
Mandible dental parameters: i to Nb (°)	24	30
Mandible dental parameters: IMPA (°)	88	92
Maxillary-mandible: Interincisal-angle (°)	127	130

**Figure 6:**
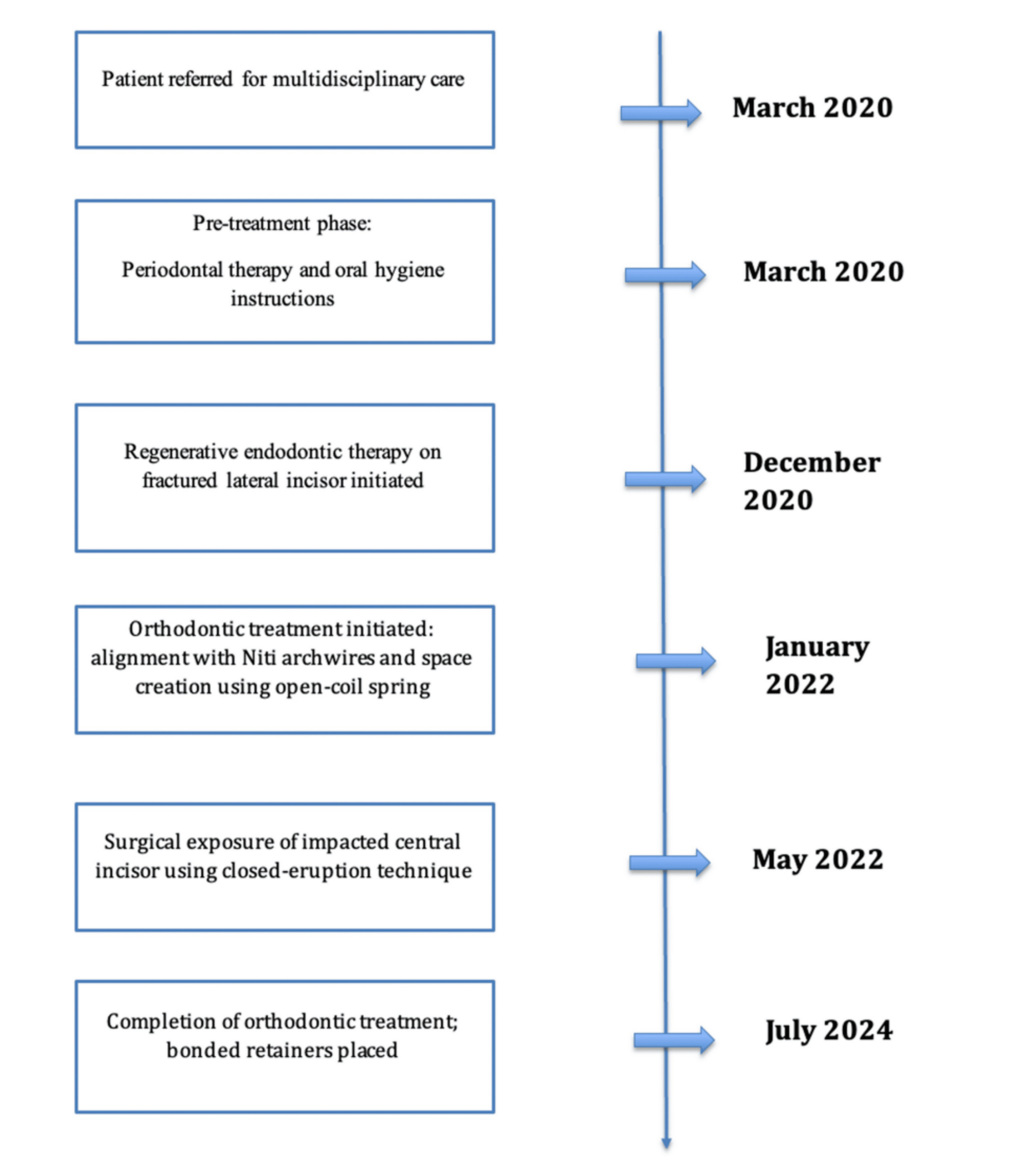
Timeline of the patient's treatment

The overall multidisciplinary management, including regenerative endodontic therapy, periodontal care, and orthodontic treatment, spanned from 2020 to 2024. The orthodontic treatment itself lasted 30 months. Bonded retainers were fixed on both the upper and lower arches. Figure 6 summarizes the timeline of the patient’s treatment. 

## Discussion

Successful management of this case was achieved by respecting the treatment protocol and ensuring a close multidisciplinary approach. Given the complexity of this case, significant challenges were present, including a skeletal Class III discrepancy, the impaction of a maxillary central incisor, and a maxillary lateral incisor with an uncertain prognosis. These conditions significantly increased the complexity of treatment planning.

Given the patient’s chief complaint and clinical parameters, the treatment plan was directed toward camouflage orthodontic treatment since the patient has an acceptable profile and only a mild skeletal Class III discrepancy. At the same time, particular attention was given to conservation and traction of the impacted maxillary incisor to restore the smile esthetics and achieve functional occlusion. Furthermore, our reflection in this case was also dictated by the prognosis of the maxillary lateral incisor, which necessitated interdisciplinary evaluation.

When treating impacted maxillary incisors, careful soft tissue management is essential to ensure a successful esthetic outcome. Two main techniques are usually described for the surgical exposure of impacted teeth: open and closed eruption. In this case report, the closed eruption technique was chosen, the flap was repositioned and sutured to its original position, mainly to ensure adequate keratinized tissue and achieve better gingival contours of the incisor. Several studies have reported better esthetic and periodontal outcomes with the closed eruption technique [6-9].

Biomechanically individualized planning and a well-controlled anchorage are important factors for the successful traction of impacted teeth. As Newton’s third law states: “For every action, there is an equal and opposite reaction”. Therefore, during the vertical extrusion movement of the impacted central incisor, a vertical intrusive force must be controlled. In addition, parasitic movements in the sagittal and transverse planes can also occur. In this case, a transpalatal bar was used to reinforce anchorage, and with the aid of a traction arm, the extrusion could be initiated even in the absence of heavy archwires. The direction of traction must be carefully evaluated, and the impacted incisor should be guided as close as possible to its normal position, while preserving adjacent teeth. Thus, to avoid crown-root collisions, a clockwise force moment was applied, allowing the crown of the impacted tooth to move away from the root of the adjacent incisor, and enabling sequential and safe traction.

For anchorage control, different strategies have been described in the literature: some authors used the remaining dentition as one single continuous anchorage unit, whereas others preferred the use of temporary skeletal anchorage devices or customized fixed palatal appliances [6,10-12].

Another challenge when treating impacted teeth is controlling root torque after traction and alignment. Torque correction can be particularly difficult, especially in cases of dilacerations of the incisor root. Several approaches exist for root torque control of a single tooth; one relies on the interaction between the wire and the bracket slot, such as bends or a high torque bracket, or employs auxiliaries such as Warren Spring. In this patient, gradual third-order bends were used in a 19x25 stainless steel archwire to place the root palatally. The bends were progressively activated in each appointment by using a 444 plier until we obtained the ideal esthetic outcome [6,11,13,14].

## Conclusions

The management of impacted maxillary incisors remains one of the most difficult cases to treat in orthodontics due to the complexity of biomechanics, the risk of collateral damage, and the esthetic and functional importance of these anterior teeth. In this case report, a multidisciplinary approach combining surgical exposure, biomechanically controlled orthodontic traction, and careful torque management allowed the successful alignment of the impacted central incisor. Treatment was completed with a stable therapeutic Class III occlusion, coincident dental midlines, and normalized overjet and overbite.

This case shows the importance of individualized treatment planning and close collaboration between specialists to ensure both functional and optimal esthetic results. Early diagnosis and intervention remain crucial to reducing the treatment complexity and improving the prognosis of similar cases.
